# Laterality of Radiation Therapy in Breast Cancer is Not Associated With Increased Risk of Coronary Artery Disease in the Contemporary Era

**DOI:** 10.1016/j.adro.2024.101583

**Published:** 2024-07-30

**Authors:** Lakshya Seth, Omar Makram, Amr Essa, Vraj Patel, Stephanie Jiang, Aditya Bhave, Sandeep Yerraguntla, Gaurav Gopu, Sarah Malik, Justin Swaby, Johnathon Rast, Caleb A. Padgett, Ahmed Shetewi, Priyanshu Nain, Neal Weintraub, Eric D. Miller, Susan Dent, Ana Barac, Rakesh Shiradkar, Anant Madabhushi, Catherine Ferguson, Avirup Guha

**Affiliations:** a Department of Medicine, Medical College of Georgia, Augusta, Georgia; b Department of Radiation Oncology at the Arthur G. James Cancer Hospital and Richard J. Solove Research Institute, The Ohio State University Comprehensive Cancer Center, Columbus, Ohio; c Duke Cancer Institute, Duke University, Durham, North Carolina; d Division of Cardio-Oncology, Inova Schar Cancer Institute and Inova Heart and Vascular Institute, Fairfax, Virginia; e Wallace H Coulter Department of Biomedical Engineering, Emory University and Georgia Institute of Technology, Atlanta, Georgia; f Department of Radiation Oncology, Medical College of Georgia, Augusta University, Augusta, Georgia; g Cardio-Oncology Program, Medical College of Georgia, Augusta, Georgia

## Abstract

**Purpose:**

External beam radiation therapy (EBRT) is a critical component of breast cancer (BC) therapy. Given the improvement in technology in the contemporary era, we hypothesized that there is no difference in the development of or worsening of existing coronary artery disease (CAD) in patients with BC receiving left versus right-sided radiation.

**Methods and Materials:**

For the meta-analysis portion of our study, we searched PubMed, Web of Science, and Scopus and included studies from January 1999 to September 2022. CAD was identified using a homogenous metric across multiple studies included. We computed the risk ratio (RR) for included studies using a random effects model. For the institutional cohort portion of our study, we selected high cardiovascular-risk patients who received diagnoses of BC between 2010 and 2022 if they met our inclusion criteria. We performed a Cox proportional hazards model with stepwise adjustment.

**Results:**

A pooled random effects model with 9 studies showed that patients with left-sided BC receiving EBRT had a 10% increased risk of CAD when compared with patients with right-sided BC receiving EBRT (RR, 1.10; 95% CI, 1.02-1.18; *P* = .01). However, subgroup analysis of 6 studies that included patients diagnosed after 1980 did not show a significant difference in CAD based on BC laterality (RR, 1.07; 95% CI, 0.95-1.20; *P* = .27). For the institutional cohort portion of the study, we found that patients with left-sided BC who received EBRT did not have a significantly higher risk of CAD when compared with their right-sided counterparts (hazard ratios [HR], 0.73; 95% CI, 0.34-1.54; *P* = .402).

**Conclusions:**

Our study suggests a historical trend of increased CAD in BC patients receiving left-sided EBRT. Data from patients diagnosed after 2010 in our institutional cohort did not show a significant difference**,** emphasizing that modern EBRT regimens are safe, and laterality of BC does not affect CAD outcomes in the short term after a BC diagnosis.

## Introduction

Breast cancer (BC) is the most common cancer diagnosis in women and the second leading cause of cancer death.^1^ The BC mortality rate has declined by 43% since 1989 because of the increased prevalence of breast screening and advancement in effective available treatments.^1^ The use of adjuvant external beam radiation therapy (EBRT) in BC treatment has resulted in decreased BC mortality; however, it has also been associated with increased incidence of cardiovascular (CV) events and mortality,^2^^,^^3^ especially in those with pre-existing CV risk factors.^4^ Research outlining the cardiotoxic effects of EBRT has led to considerable decreases in radiation dosages delivered to the heart in modern regimens. Despite this advancement, mean doses to the heart still range between 2 and 7 Gy, depending on the laterality of BC.^5^^,^^6^ The mean heart dose has been noted to be higher in left-sided BC and that has been associated with increased coronary artery disease (CAD) events in women with left-sided BC.^4^^,^^7^ In order to minimize unintended cardiotoxicity, modern radiation techniques have evolved since the 1980s^8^ to minimize radiation to the heart without decreasing clinical effectiveness.^9^ Techniques such as deep inspiration breath hold (DIBH) and intensity modulated radiation therapy aim to minimize the radiation dose to peripheral organs such as the heart^10^, ^11^, ^12^, ^13^, ^14^, ^15^, ^16^, ^17^, ^18^, ^19^ without compromising the radiation dose to the breast^19^, ^20^, ^21^, ^22^ and have been proven to be effective.

Given the improvement in radiation delivery technology, we hypothesized that there is no difference in the development of/worsening of CAD in patients with BC receiving left versus right-sided radiation. To answer this question, we conducted an updated meta-analysis to include more contemporary data.^6^^,^^23^, ^24^, ^25^, ^26^, ^27^, ^28^, ^29^, ^30^ There are significant discrepancies in the technology for BC radiation among studies, lack of adjustment for preventive CV care in the current decade, and lack of data regarding mean/median heart dose in the cohort studies we included in the meta-analysis. Thus, we supplemented this meta-analysis with a 10-year single-institution data set between 2010 and 2022 to mitigate these issues.

## Methods and Materials

### Meta-analysis

#### Literature study strategy

This meta-analysis was conducted in accordance with the Preferred Reporting Items for Systematic Reviews and Meta-Analyses guidelines.^31^ We performed the systematic literary search in August of 2022 and encompassed 3 databases: PubMed, Web of Science, and Scopus. The search strategy consisted of keywords and standardized MeSH terms as well as Boolean operators (“OR” or “AND”). Keywords included “Right or Left” AND “Coronary Artery Disease OR Ischemic Heart Disease OR Cardiac Events OR Cardiovascular Events OR Morbidity” AND “Radiotherapy” AND “Breast Cancer OR Breast Neoplasms.” No restrictions were used.

#### Inclusion criteria

Studies were included if they met the following criteria: (1) published in English; (2) women with BC were the study population; (3) reported CAD events comparing left-sided versus right-sided EBRT in patients with BC; (4) CAD was defined using International Classification of Diseases 9th edition codes 410 to 414 or 10th edition codes I20 to I25; (5) the 2 groups being compared were patients with left-sided BC receiving EBRT and patients with right-sided BC receiving EBRT.

#### Data extraction and quality assessment

Four blinded reviewers participated in the abstract and full-text screenings. Conflicting decisions were resolved by collaboration between 2 reviewers (L.S. and A.E.). For each selected study, the following data were extracted: author's name, year of publication, geographic location, study type, duration of follow-up, type/stage of BC, radiation protocols, adjuvant therapy, risk factors, number of events, and number of patients. The quality of the included studies was determined by 2 reviewers (L.S. and J.S.) using the New Castle Ottawa scale for cohort studies^32^ (Table E1**)**.

#### Statistical analysis

A random effects model was used to calculate the risk ratio (RR) with a 95% CI. DerSimonian-Laird method was used for estimating between-study variance and fitting the random-effect model. Heterogeneity was assessed using Cochran's Q static and quantified using Higgins I-square statistic.^33^ Publication bias and small-study effects were assessed graphically through funnel plots and statistically through regression-based Egger test. A sensitivity analysis using trim and fill analysis was conducted to assess the robustness of the results. A forest plot was used to graphically present the RR of each study as well as pooled RR. A *P* value of <.05 was considered statistically significant. IBM SPSS and STATA/MP 17.0 (StataCorp) software were used for statistical analysis.

### Institutional data set

Patients were selected sequentially from a cohort of patients who received diagnoses of BC between 2010 and 2022 in our institution where EBRT was used on only one side of the chest. This study was approved by the Augusta University Institutional Review Board.

#### Inclusion criteria

Patients were included if they met the following criteria:

1. Were considered high CV risk prior to treatment based on prior myocardial infarction/stroke or having 3 or more of the following risk factors: age >55 years, hypertension, diabetes, high cholesterol, tobacco use, and family history of early CV disease. The higher risk cohort was a priori selected because the event rates in lower-risk cohorts are <1% annually making institutional sensitivity analysis not feasible because of the required sample size.^34^
2. Received diagnoses of unilateral BC after 2010
3. Did not receive diagnoses of another primary malignancy
4. Received EBRT as part of their treatment regimen
5. Had an accurate record of risk factor data including pre-existing conditions and medications before the date of diagnosis
6. Had accurate information regarding the type and stage of BC and detailed information regarding the treatment protocol as defined by radiation dosage information and chemo/endocrine/immunotherapy the patient was receiving.

#### Data extraction

Nine researchers participated in data extraction and collected pertinent patient information from electronic medical records. For each patient, the following data were extracted: demographic information (name, medical record number, gender, race, date of birth, body mass index [BMI], and age at diagnosis), prior risk factors (history of hypertension, hypercholesterolemia, smoking, diabetes, heart failure, atrial fibrillation, atrial flutter, myocardial infarction, cerebrovascular accident or transient ischemic attack, renal function, CAD, peripheral artery disease, chronic obstructive pulmonary disease, rheumatologic disease, hypothyroidism, and depression), cancer information (date of diagnosis, laterality, stage, ductal, tumor/node/metastasis status, ER/PR/HER2 status, date of chemotherapy, date of radiation therapy, date of hormone therapy, date of immunotherapy, type of radiation, total radiation dose, HER2 agents, and type of surgery), baseline vitals, laboratory tests and imaging at cancer diagnosis (BMI, systolic blood pressure, diastolic blood pressure, heart rate, chest computed tomography, ejection fraction, global longitudinal strain, atrial size, potassium, aspartate aminotransferase, alanine transaminase, creatinine, glucose, hemoglobin, neutrophil count, lymphocyte count, white blood cell count, C-reactive protein, A1c, total cholesterol, triglycerides, high density lipoprotein, and allostatic load) and CAD events (myocardial infarction/ST-elevation myocardial infarction/non ST-elevation myocardial infarction/unstable angina, percutaneous coronary intervention without myocardial infarction, and coronary artery bypass graft) and vitals, laboratory tests, and imaging at the time of first CAD event (BMI, systolic blood pressure, diastolic blood pressure, heart rate, chest computed tomography, ejection fraction, global longitudinal strain, atrial size, potassium, aspartate aminotransferase, alanine transaminase, creatinine, glucose, hemoglobin, neutrophil count, lymphocyte count, white blood cell count, C-reactive protein, A1c, total cholesterol, triglycerides, high density lipoprotein, and allostatic load).

#### Statistical analysis

The data were presented as absolute values and percentages for categorical variables and as median and IQR for continuous variables. Categorical variables were compared using Pearson's χ^2^ test. Data distribution assumptions for continuous variables were confirmed using histograms and the Kolmogorov-Smirnov test, followed by paired samples *t* test for normally distributed variables and nonparametric Mann-Whitney *U* tests for nonnormal distributed variables. To investigate the association between the laterality of BC and CAD, we tested for proportional hazards assumption, then conducted the Cox proportional hazards model with stepwise adjustment and presented the results as hazard ratios (HRs). After presenting the unadjusted model, we adjusted for age and race (model 1). In the subsequent models, we sequentially adjusted for diabetes, hypertension, hypercholesterolemia, BMI, smoking status, and chronic kidney disease (model 2), for CAD (model 3), for cancer stage, type of surgery, radiation dose, hormone therapy, chemotherapy, immunotherapy, and use of HER-2 agents (model 4), and DIBH (model 5 as sensitivity analysis). We additionally conducted a subgroup analysis based on prior CAD versus no prior CAD. A *P* value of <.05 was considered statistically significant. IBM SPSS and STATA/IC 16.1 (StataCorp) software were used for statistical analysis.

## Results

### Demographics

#### Meta-analysis

**Figure 1:**
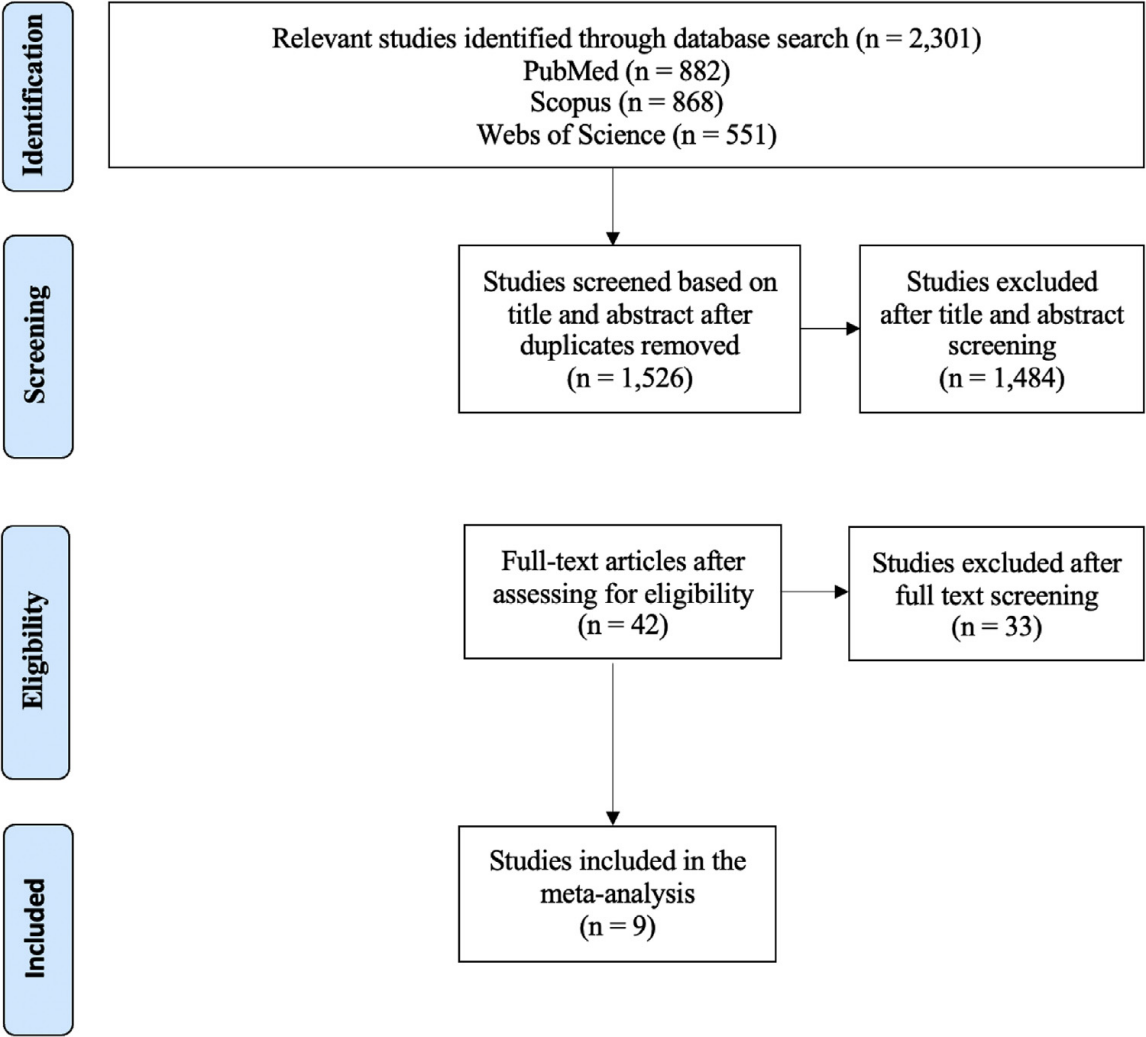
Preferred Reporting Items for Systematic Reviews and Meta-Analyses diagram of the meta-analysis of the risk of coronary artery disease events in breast cancer patients receiving left-sided external beam radiation therapy versus right-sided external beam radiation therapy.

A total of 2301 studies were retrieved from the initial literature search, of which 775 were duplicates and removed. Of the 1526 studies remaining, 42 were included for full-text screening. Of the 42 studies analyzed, 33 were excluded after full-text screening (Fig. 1). A total of 9 studies^6^^,^^23^, ^24^, ^25^, ^26^, ^27^, ^28^, ^29^, ^30^ were included in the final meta-analysis, involving 118,643 patients (60,947 left-sided and 57,696 right-sided patients) and 6211 CAD events (3347 left-sided events and 2864 right-sided events). Most of the studies analyzed included patients in the age range of 50 to 65 years. Median follow-up time was 11.1 years (Table 1).^6^^,^^23^^-^^30^

**Table 1:** Baseline characteristics of the studies included in the meta-analysis

Author	Country	Type of study	Type of breast cancer	No. of patients	Date of breast cancer diagnosis
Boekel et al,^23^ 2014	Netherlands	Retrospective cohort	Ductal carcinoma in situ	L: 1263 R: 1061	1989-2004
Borger et al,^24^ 2007	Netherlands	Retrospective cohort	T1-2N0	L: 870 R: 731	1980-1993
Højris et al,^25^ 1999	Denmark	Retrospective cohort	Nonmetastatic	L: 755 R: 770	1982-1990
Hooning et al,^26^ 2007	Netherlands	Retrospective cohort	Stage I, II, or IIIa	L: 1837 R: 1725	1970-1986
McGale et al,^6^ 2011	Denmark/ Sweden	Prospective cohort	All breast cancer diagnoses	L: 17912 R: 16913	1976-2006
Patt et al,^27^ 2005	United States	Retrospective cohort	In situ, localized or regional stage	L: 8363 R: 7907	1986-1993
Rehammar et al,^28^ 2017	Denmark	Retrospective cohort	All breast cancer diagnoses	L: 9915 R: 9549	1977-2005
Wadsten et al,^29^ 2018	Sweden	Retrospective cohort	Ductal carcinoma in situ	L: 1242 R: 1188	1992-2012
Wennstig et al,^30^ 2020	Sweden	Retrospective cohort	All invasive breast cancer diagnoses	L: 18790 R: 17852	1992-2012

#### Institutional data set

A total of 2692 patients received diagnoses of BC in our institution between January 1, 2010, and January 1, 2022, of whom 1894 patients received EBRT as part of their treatment. Of these, 222 patients with left-sided BC and 245 patients with right-sided BC met the inclusion criteria and were included in the final analysis. The median age at diagnosis was 65 years (IQR, 60-72 years) for patients with left-sided BC and 65 years (IQR, 59-72 years) for patients with right-sided BC. The prevalence of hypertension (92.34% vs 90.20%, *P* = .415), hypercholesterolemia (59.46% vs 58.61%, *P* = .852), and diabetes (44.59% vs 40.41%, *P* = .361) was similar in patients with left-sided versus right-sided BC. There was no significant difference in all-cause mortality (*P* = .164), or CV mortality (*P* = .727) based on laterality of BC. The median total radiation dose was 5680 cGy (IQR, 5256-9860 cGy) in patients with left-sided BC and 6040 cGy (IQR, 5256-10,080 cGy) in patients with right-sided BC, with no significant difference in dosage based on laterality of BC (*P* = .604) (Table 2). The median follow-up time was 2.58 years.

**Table 2:** Baseline characteristics and CAD events from our institutional cohort

Variables	Patients with left-sided breast cancer (n = 222)	Patients with right-sided breast cancer (n = 245)	*P* value⁎
Age at diagnosis, median (IQR), y	65 (60-72)	65 (59-72)	.895
Ethnicity			
White	46.40	50.61	.363
Black	48.20	46.12	.654
Asian	3.15	2.45	.644
Hispanic	1.35	0.41	.350†
BMI at diagnosis, median (IQR), kg/m^2^	30.8 (27.2, 36.7)	31 (26.45, 38.05)	.821
Smoking history			
Current	14.41	18.78	.207
Former	16.67	16.33	.921
None	68.92	64.90	.357
Prior health conditions			
Hypertension	92.34	90.20	.415
Diabetes mellitus	44.59	40.41	.361
Hypercholesterolemia	59.46	58.61	.852
Chronic kidney disease	4.95	5.31	.864
Heart failure	4.50	4.08	.822
Atrial fibrillation	4.95	3.27	.356
Atrial flutter	0.45	0.41	1.000†
Myocardial infarction	5.86	6.12	.904
Clinical CAD	10.36	12.65	.439
PAD	1.35	2.04	.727†
CVA/TIA	8.56	6.53	.406
COPD	7.66	4.49	.150
Rheumatologic disease	13.96	18.78	.162
Hypothyroidism	15.32	18.78	.322
Depression	13.06	11.89	.700
Cancer stage			
Ductal carcinoma in situ	15.77	12.65	.335
I	53.15	53.47	.945
II	21.62	23.67	.597
III	8.11	7.76	.888
IV	0.90	1.22	1.000†
N/A	0.45	1.22	.625†
Receptor status			
ER+	82.43	83.27	.741
PR+	79.19	78.78	.950
HER2+	15.77	15.92	.935
Cancer therapy			
Hormone therapy	69.37	71.84	.567
Immunotherapy	11.26	9.80	.594
Chemotherapy	35.45	42.04	.146
Use of HER-2 agents	14.86	15.51	.832
Surgery			
Mastectomy	22.97	20.82	.573
Lumpectomy	77.03	78.37	.728
None	0.00	0.82	.500†
Use of DIBH			
Breath hold	41.44	0.00	<.001†, ⁎
Free breath	45.50	86.94	<.001⁎
Unknown	13.06	13.06	1.000
Nodal coverage			
Tangents	59.00	58.37	.888
Comprehensive	6.76	4.08	.200
Other‡	23.87	23.27	.877
Unknown	10.36	14.29	.199
Total radiation dose, median (IQR), in cGy	5680 (5256-9860)	6040 (5256-10,080)	.096
Incidence of CAD events	4.95	7.35	.285
CAD event			
Myocardial infarction NSTEMI STEMI Unstable angina	4.05	4.08	.988
PCI without MI	0.90	2.04	.454†
CABG	0.45	1.22	.625†
Death	5.41	2.86	.164
CV death	1.35	2.04	.727†

*Abbreviations:* BMI = body mass index; CABG = coronary artery bypass graft; CAD events = coronary artery disease events; COPD = chronic obstructive pulmonary disease; CV = cardiovascular; CVA = cerebrovascular accident; DIBH = deep inspiration breath hold; MI = myocardial infarction; NSTEMI = non–ST-elevation myocardial infarction; PAD = peripheral artery disease; PCI = percutaneous coronary intervention; STEMI = ST-elevation myocardial infarction; TIA = transient ischemic attack.

⁎ Signifies a statistically significant p value (p < 0.05).

† A 2-sided Fisher exact test was used to obtain this *P* value because the χ^2^ assumption was violated because of fewer than 5 expected counts in >1 cell.

‡ Other is defined as a category comprising patients who in addition to tangential treatment, received radiation to subclavian nodes, posterior axillary boost, or just received partial radiation. Values are percentages unless noted otherwise.

### Outcomes

#### Meta-analysis

**Figure 2:**
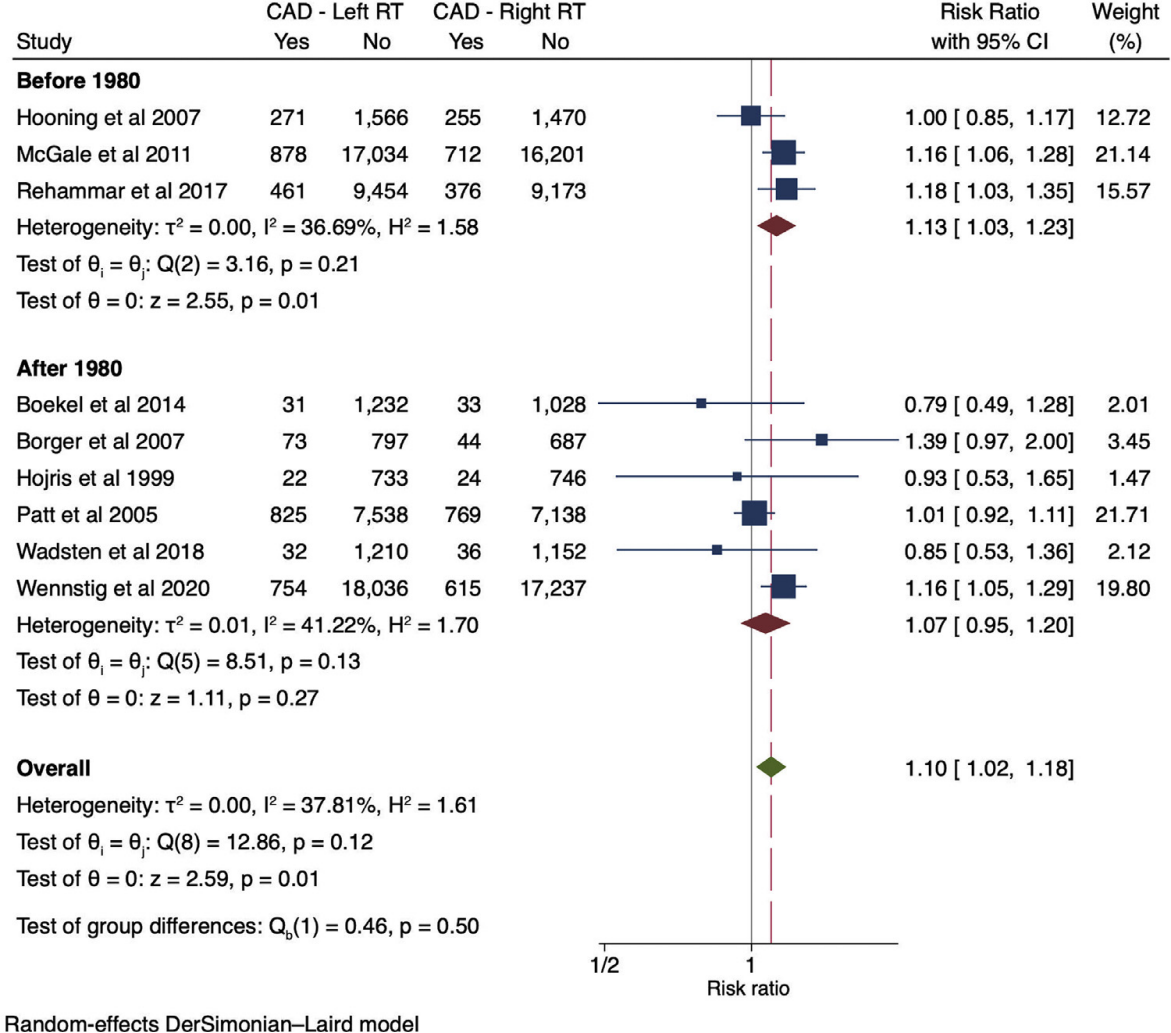
Forest plot for the risk of coronary artery disease events in patients with breast cancer receiving left-sided external beam radiation therapy versus right-sided external beam radiation therapy.^6^^,^^23^, ^24^, ^25^, ^26^, ^27^, ^28^, ^29^, ^30^ *Abbreviations:* CAD = coronary artery disease; RR = relative risk.

A pooled random effects model of 9 studies showed that patients with left-sided BC receiving EBRT had a 10% increased risk of CAD when compared with patients with right-sided BC receiving EBRT (RR, 1.10; 95% CI, 1.02-1.18; *P* = .01) (Fig. 2).^6^^,^^23^, ^24^, ^25^, ^26^, ^27^, ^28^, ^29^, ^30^ A subgroup analysis of 6 studies that included patients who received diagnoses of BC after 1980 did not show a statistically significant difference in CAD based on laterality of BC (RR, 1.07; 95% CI, 0.95-1.20; *P* = .27) (Fig. 2). Visual inspection of the funnel plot displayed risk for publication bias; hence, a trim and fill approach was used to adjust for the risk (Fig. E1). The trim and fill random effects model added 2 studies and showed an 11% increased risk of CAD in patients with left-sided BC receiving EBRT (RR, 1.11; 95% CI, 1.04-1.19; *P* = .002).

#### Institutional data set

The primary outcome of CAD was noted in 4.95% of patients with left-sided BC and 7.35% of patients with right-sided BC, with no significant difference in incidence based on laterality of BC (*P* = .284) in totality or based on individual CAD event (Table 2). In an unadjusted model, patients with left-sided BC did not have a significantly higher risk for CAD when compared with patients with right-sided BC (HR, 0.73; 95% CI, 0.34-1.54; *P* = .402) (Table 3). Similar results were found in subsequent models, including the fully adjusted model (model 4; adjusted hazards ratio, 0.44; 95% CI, 0.06-3.27; *P* = .420). Among patients with no prior CAD, an unadjusted model showed that patients with left-sided BC did not have a significantly higher risk for CAD when compared with patients with right-sided BC (HR, 0.65; 95% CI, 0.22-1.95; *P* = .445) (Table 3). Similar results were found in subsequent models. Among patients with prior CAD, an unadjusted model showed that patients with left-sided BC did not have a significantly higher risk for CAD when compared with patients with right-sided BC (HR, 0.99; 95% CI, 0.35-2.78; *P* = .980). Similar results were found in subsequent models, including when adjusting for DIBH (Table 3, Table E2).

**Table 3:** Cox proportional hazards model for the outcome of CAD in the entire cohort, those with no prior CAD and those with prior CAD

				HR (95% CI, *P* value)				
Outcome	Radiation side	Events/total*	Time at risk (person-months)	Univariable model	Model 1	Model 2	Model 3	Model 4
All population (n = 467)								
CAD event	Right (n = 245)	29/467	1680.7	Reference	Reference	Reference	Reference	Reference
	Left (n = 222)			0.73 (0.34-1.54, *P* = .402)	0.74 (0.35-1.58, *P* = .435)	0.88 (0.30-2.63, *P* = .822)	0.98 (0.28-3.43, *P* = .970)	0.44 (0.06-3.27, *P* = .420)
No prior CAD (n = 407)								
CAD event	Right (n = 211)	14/407	1510.17	Reference	Reference	Reference	-	Reference
	Left (n = 196)			0.65 (0.22-1.95, *P* = .445)	0.76 (0.25-2.28, *P* = .624)	0.85 (0.26-2.79, *P* = .790)	-	0.58 (0.14-2.36, *P* = .450)
Prior CAD (n = 60)								
CAD event	Right (n = 34)	15/60	170.57	Reference	Reference	Reference	-	Reference
	Left (n = 26)			0.99 (0.35-2.78, *P* = .980)	0.66 (0.20-2.14, *P* = .485)	0.61 (0.14-2.63, *P* = .508)	-	0.01 (0.000005–7.43, *P* = .160)

*Abbreviations:* CAD = coronary artery disease.

⁎ Based on the univariable analysis. Model 1: Adjusted for age and race. Model 2: Model 1 + diabetes, hypertension, hypercholesterolemia, body mass index, smoking status, chronic kidney disease. Model 3: Model 2 + prior CAD. Model 4: Model 3 + cancer stage, type of surgery, radiation dose, hormone therapy, immunotherapy, chemotherapy, use of HER2.

## Discussion

This study used contemporary single-institution data along with an updated meta-analysis to determine CAD outcomes in patients who received EBRT for BC based on laterality. Although our meta-analysis demonstrated that patients with left-sided BC were more likely to develop CAD, a more contemporary subgroup analysis of patients diagnosed after 1980 did not show any association between the laterality of BC and CAD outcomes. Our single institutional cohort containing patients diagnosed after 2010 confirmed this finding while also mitigating the limitations of the meta-analysis. We conclude that modern EBRT does not affect CAD outcomes after a BC diagnosis in the short term, even among those with prior CAD.

Our study contributes to the rapidly evolving field of cardio-oncology, which aims to investigate the CV risk associated with cancer therapies and implement treatment strategies to minimize this risk in patients with cancer The findings from this study provide an updated analysis of modern radiation techniques demonstrating no difference in CAD in patients with left-sided versus right-sided BC. Literature in the field has shown that modern radiation techniques such as DIBH and intensity modulated radiation therapy along with the utilization of CT-guided treatment regimens^35^ have allowed for a more targeted organ approach and have spared the delivery of high doses of radiation to the heart.^8^^,^^36^ Findings from our institutional cohort reflect these advances, because we found that in the contemporary era, there is no difference in total radiation dose based on the laterality of treatment, which has translated into no difference in risk of CAD based on laterality of EBRT in BC therapy.

Over the past decade, there has been increased emphasis on preventative CV care in patients with cancer receiving cardiotoxic therapy. Prominent international organizations such as the American Society of Clinical Oncology, American Heart Association, European Society for Medical Oncology, and the European Society of Cardiology have all released recommendations advising screening for CV risk factors prior to initiation of cancer therapy and continued surveillance for CV events during and following completion of treatment.^37^, ^38^, ^39^, ^40^ Multidisciplinary cancer care has been established as an essential part of cancer care^41^^,^^42^ since 1995^42^ and has given rise to the rapidly growing field of cardio-oncology.^43^^,^^44^ International guidelines outlining the cardiotoxic effects of cancer therapy^37^, ^38^, ^39^, ^40^ as well as the increased number of cancer survivors^37^, ^38^, ^39^^,^^45^ has led to research outlining the key role of cardio-oncology services^43^^,^^45^, ^46^, ^47^ in managing the CV needs of patients currently receiving cancer treatment. By designing our cohort to include patients who received diagnoses of BC after 2010, we aimed to assess whether the increased implementation of preventive care has had a positive effect on CV outcomes. Although the meta-analysis portion of our study and other meta-analyses^48^^,^^49^ that include patients who received diagnoses of BC in the late 20th century have found an increased CV risk in patients with left-sided BC receiving EBRT, our updated institutional cohort found that in the contemporary era, no such difference in risk occurs. Regardless of the findings of this study, it is evident that real-world patients with BC receiving EBRT have a high burden of CV risk factors and would benefit from cardio-oncology screening and management as per guidelines.^40^^,^^50^

This meta-analysis had several limitations. First, the data were based on observational studies rather than randomized control trials. Because the observational data were sourced from country-wide databases, individual patient data were difficult to obtain. Additionally, individual radiation dose information was not available; hence, we could not investigate whether the difference in incidence of CAD events was because of increased radiation dose in left-sided BCs. These limitations are partly offset by the inclusion of the institutional data set. Using this data set allowed us to analyze individual patient information such as demographic information, prior health conditions, cancer information, and radiation information. Additionally, we were able to filter this institutional data set to only include patients diagnosed after 2010 to accurately reflect advances in modern EBRT regimens and implementation of preventative CV care in patients with cancer and analyze its subsequent effect on CAD events. Limitations of the institutional data set include incomplete or unavailable patient information and inconsistent follow-up for some patients. Additionally, our follow-up time was 2.58 years, limiting our ability to draw conclusions about the long-term cardiac effects of radiation.

## Conclusion

Our study suggests a historical trend of increased CAD in BC patients receiving left-sided EBRT. However, data from patients diagnosed after 2010 within our institutional cohort did not show a significant difference**,** emphasizing the potential of modern EBRT regimens, particularly DIBH, to offer a safer alternative. Notably, our findings suggest that the laterality of BC does not significantly affect CAD outcomes in the short term after a BC diagnosis.

## Appendix Supplementary materials

Supplementary Figures Laterality BC Project Image, application 1

## Footnotes

Supplementary material associated with this article can be found in the online version at doi:10.1016/j.adro.2024.101583.
