# Supplementary material for: Laterality of Radiation Therapy in Breast Cancer is Not Associated With Increased Risk of Coronary Artery Disease in the Contemporary Era
Source: Adv Radiat Oncol. 2024 Jul 30;9(10):101583. doi: 10.1016/j.adro.2024.101583 (PMC11385753; doi:10.1016/j.adro.2024.101583)
Supplement: Supplementary Figures Laterality BC Project [file mmc1.docx]

|  | **Selection** | | | | **Comparability** | **Outcome** | | | **Scores** |
| --- | --- | --- | --- | --- | --- | --- | --- | --- | --- |
| **Study** | **Exposed Cohort- Representativeness** | **Non-Exposed Cohort - Selection** | **Establishment of Exposure** | **Outcome of Interest Not Initially Present** | **Comparability Based on Design/Analysis** | **Assessment of Outcome** | **Sufficiently Long Follow-Up** | **Adequacy of Follow-Up** |  |
| Boekel et al, 2014 | 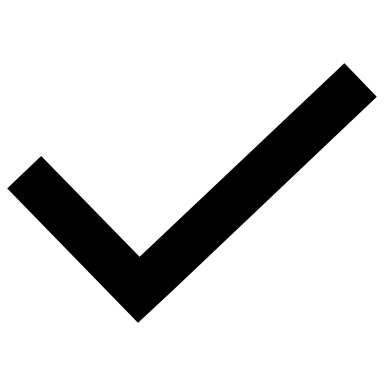 | 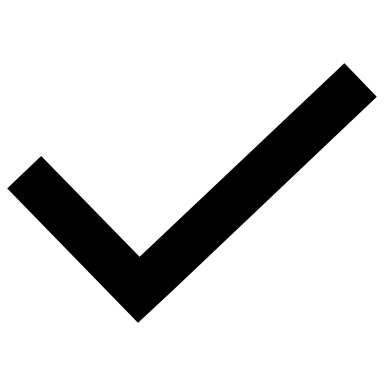 | 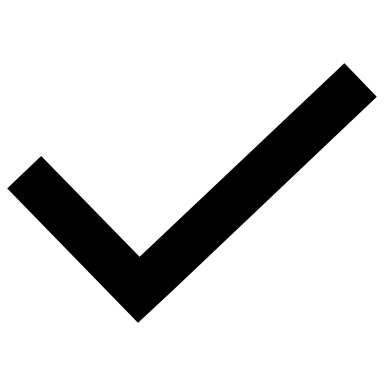 |  | 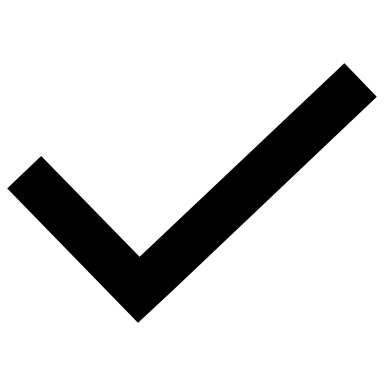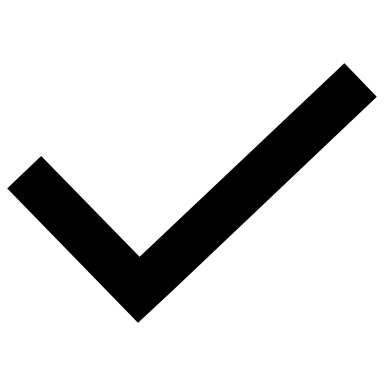 | 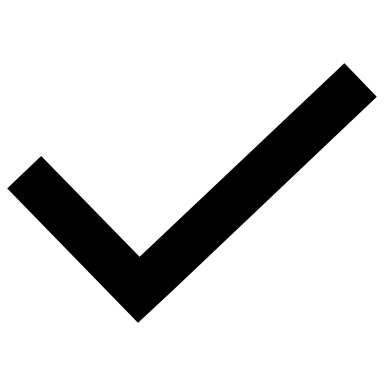 | 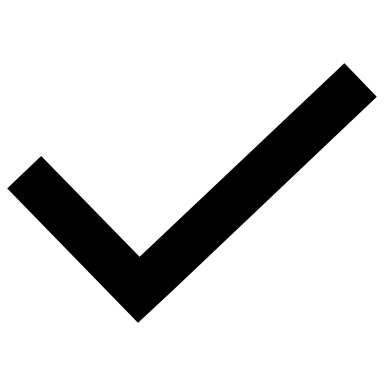 |  | 7 |
| Borger et al, 2007 | 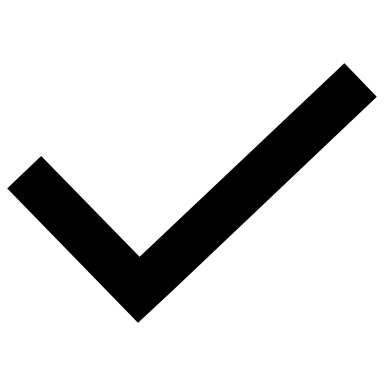 | 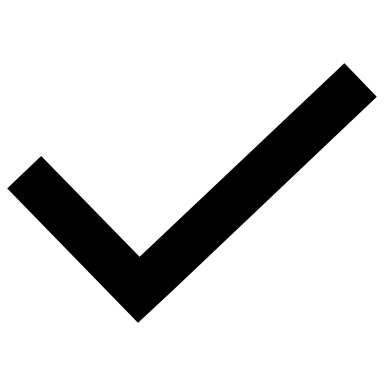 | 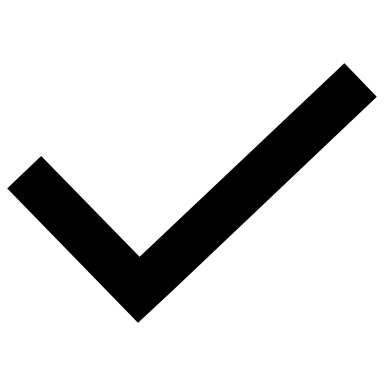 | 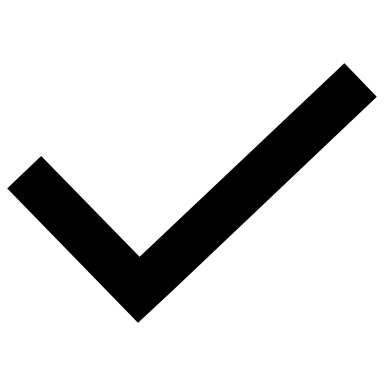 | 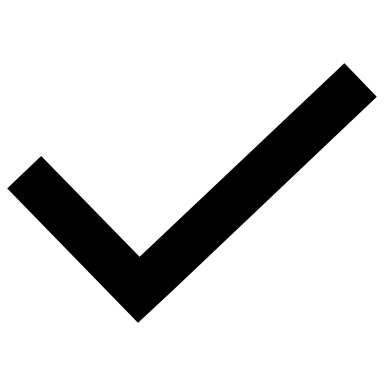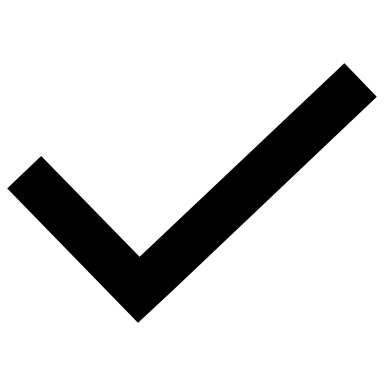 | 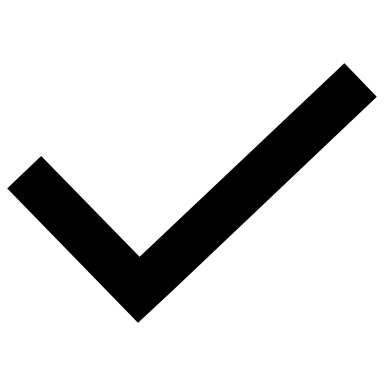 | 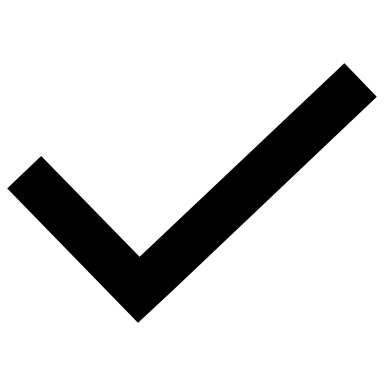 | 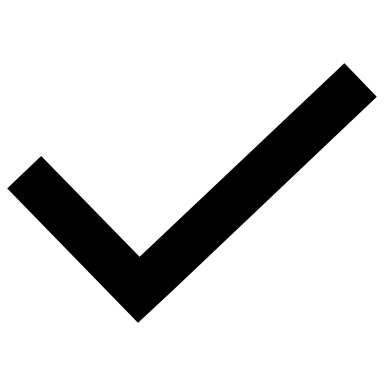 | 9 |
| Hojris et al, 1999 | 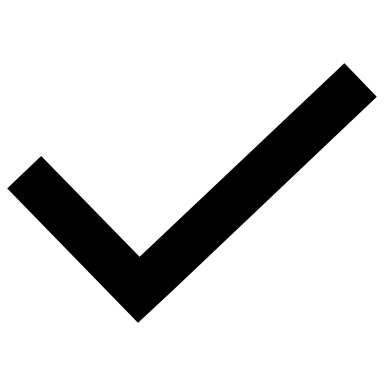 | 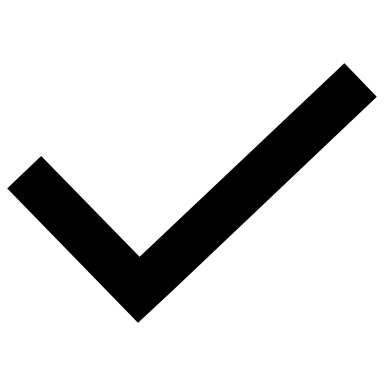 | 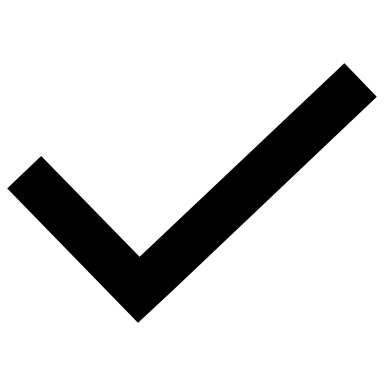 | 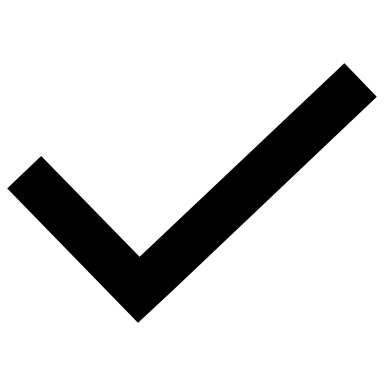 | 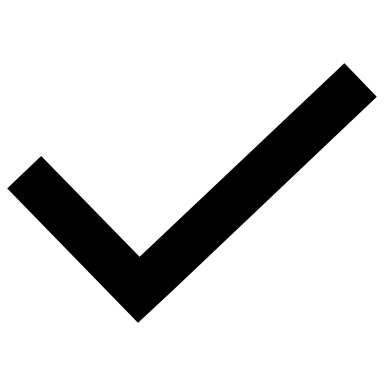 | 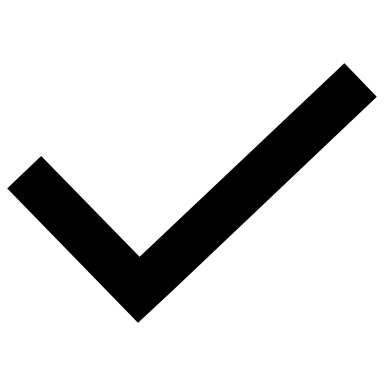 | 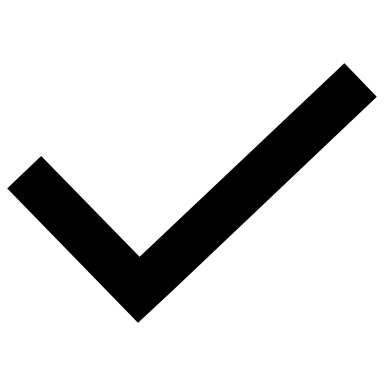 | 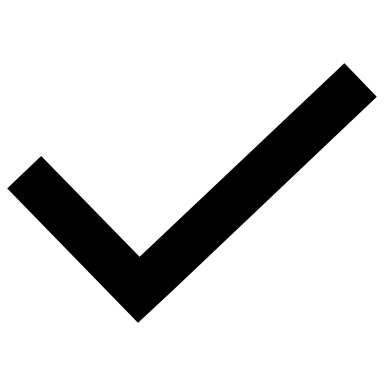 | 8 |
| Hooning et al, 2007 | 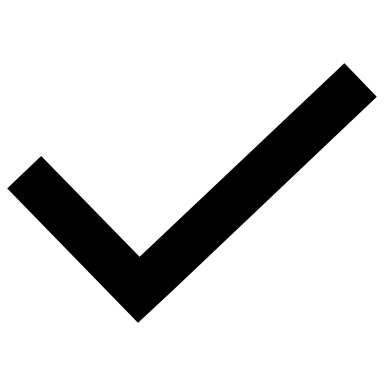 | 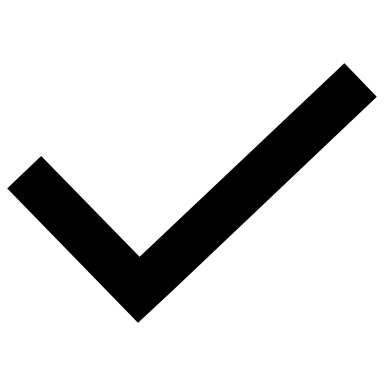 | 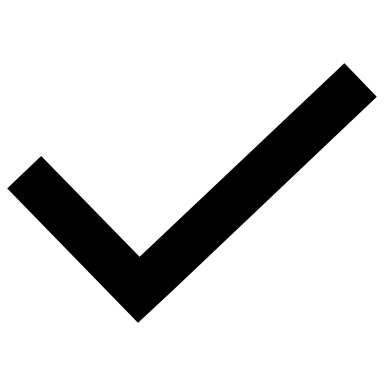 | 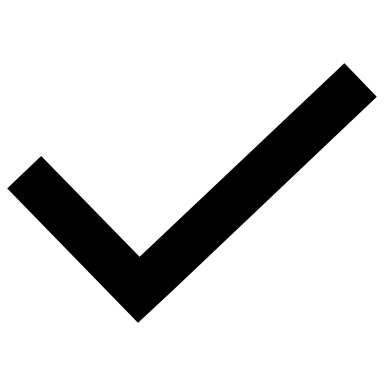 | 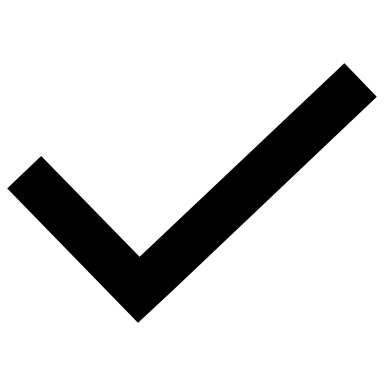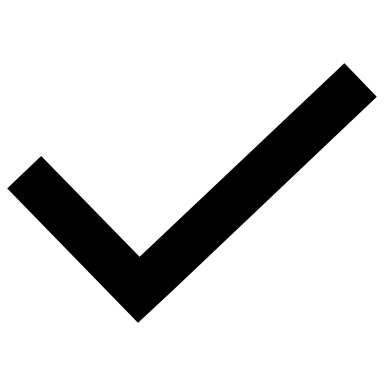 | 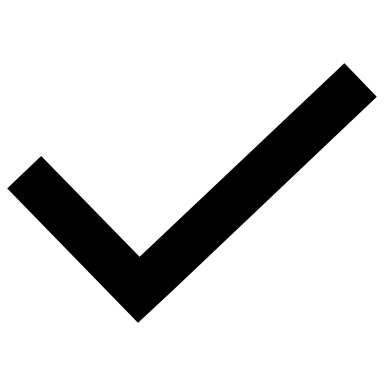 | 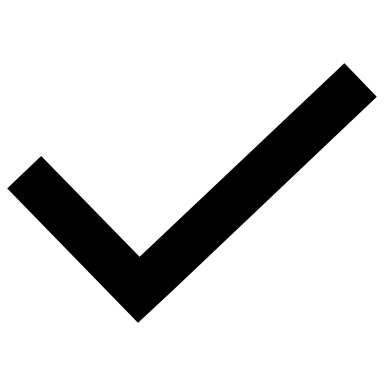 | 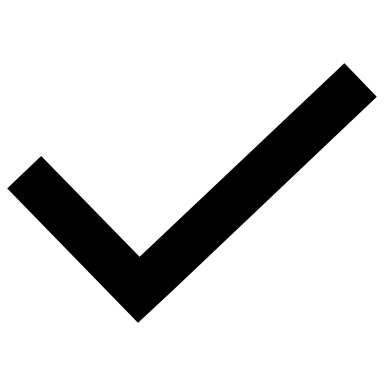 | 9 |
| McGale et al, 2011 | 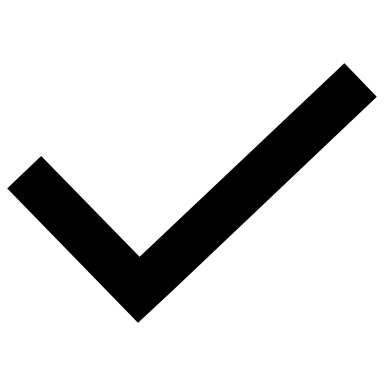 | 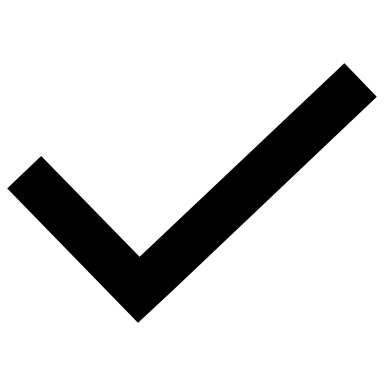 | 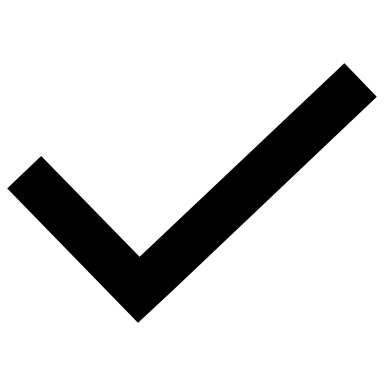 | 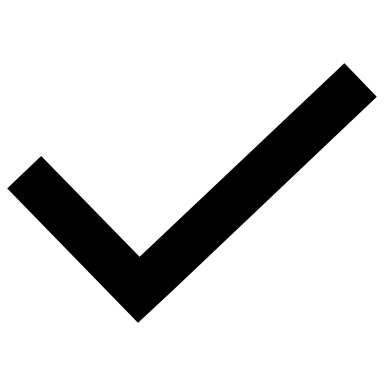 | 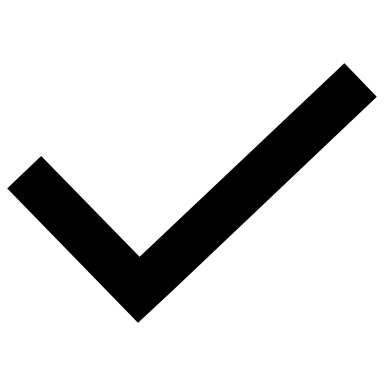 | 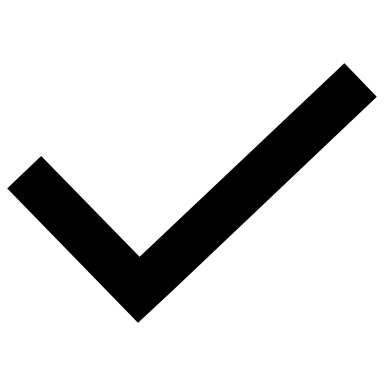 | 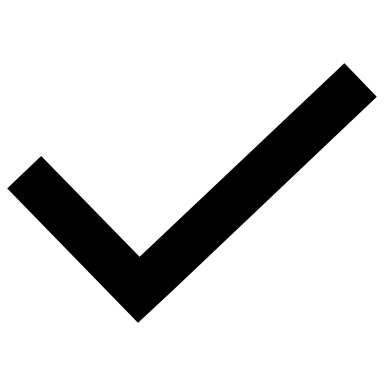 | 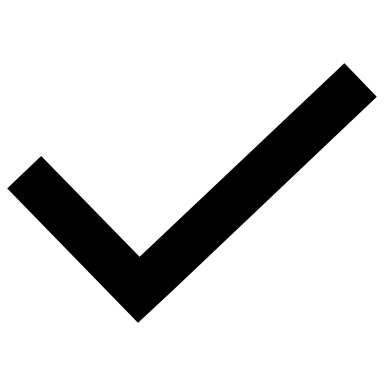 | 8 |
| Patt et al, 2005 | 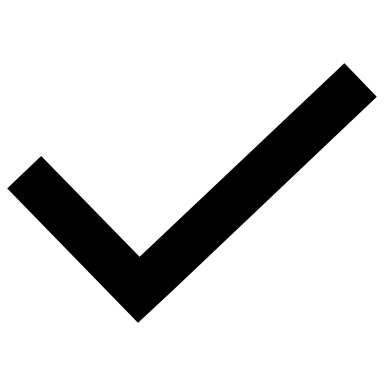 | 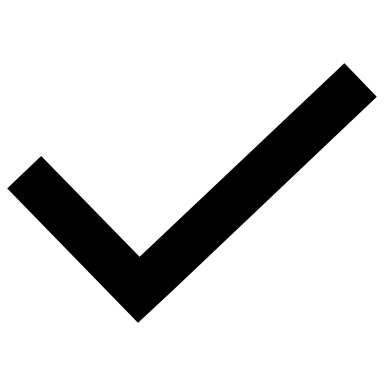 | 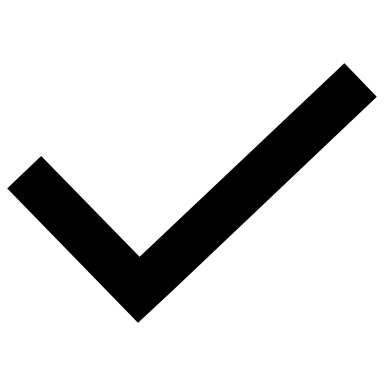 |  | 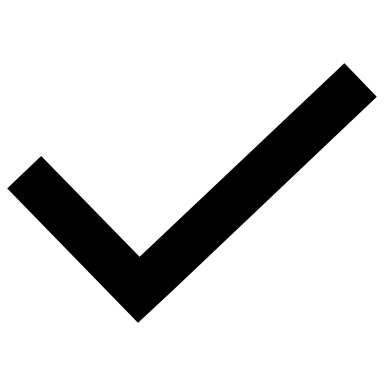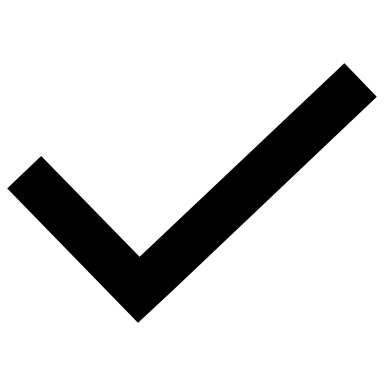 | 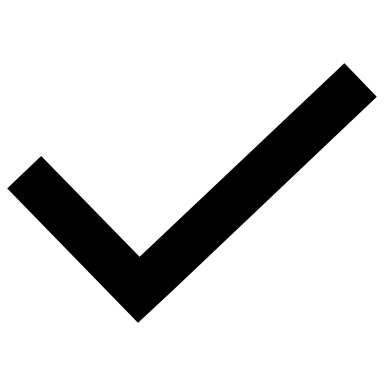 | 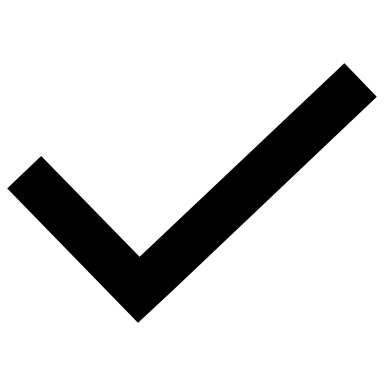 | 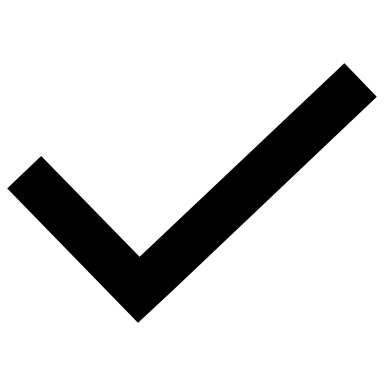 | 8 |
| Rehammar et al, 2017 | 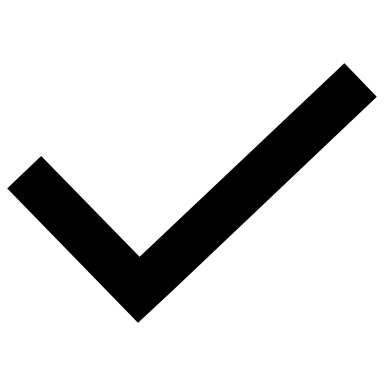 | 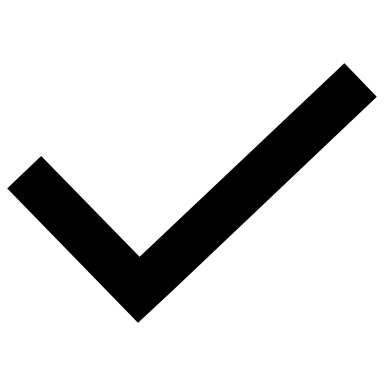 | 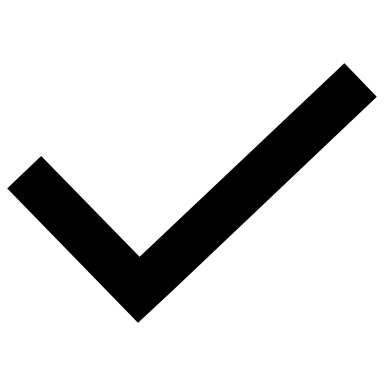 |  | 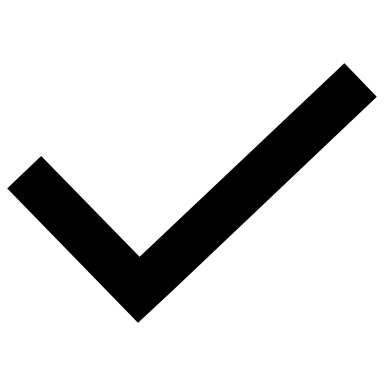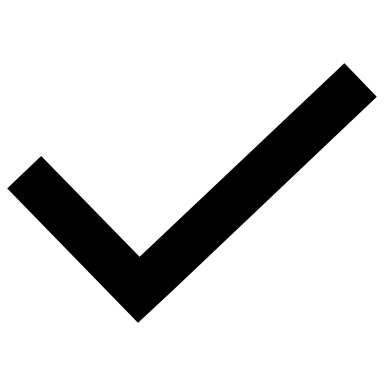 | 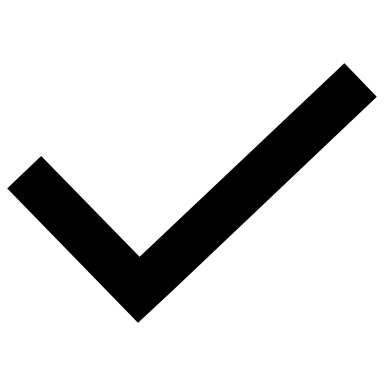 |  | 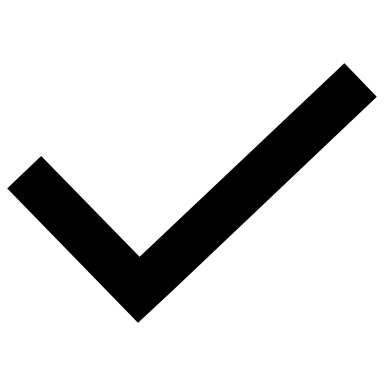 | 7 |
| Wadsten et al, 2018 | 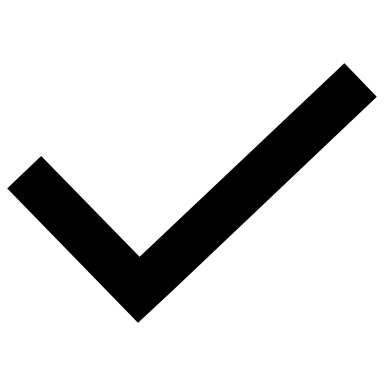 | 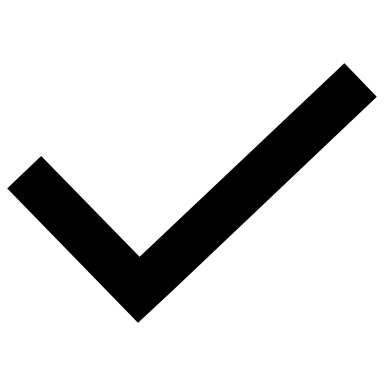 | 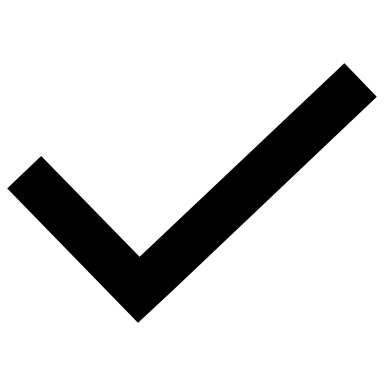 |  | 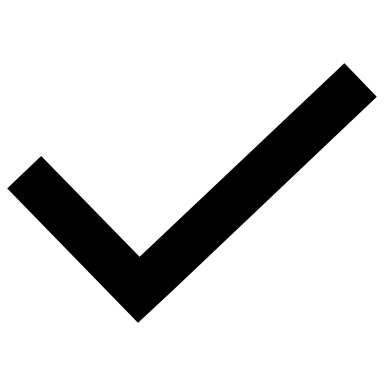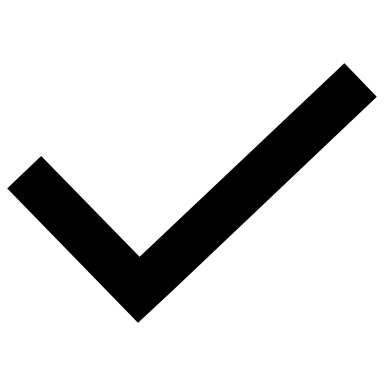 | 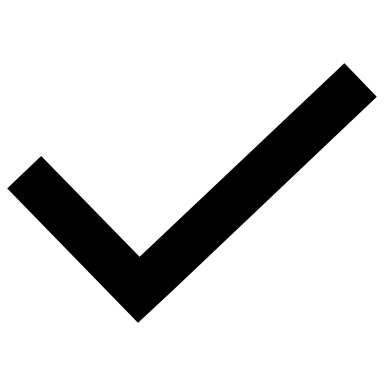 | 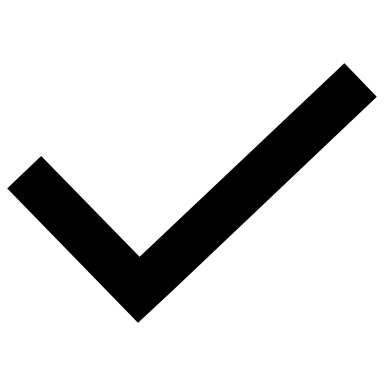 |  | 8 |
| Wennstig et al, 2020 | 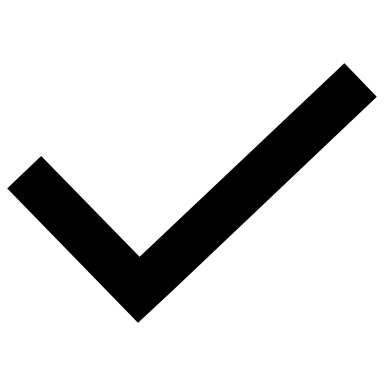 | 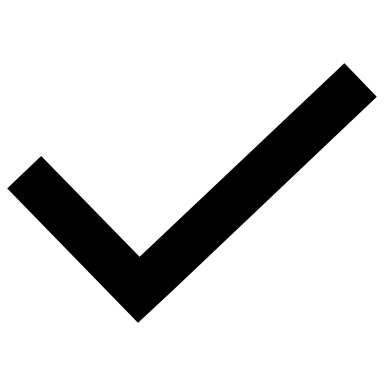 | 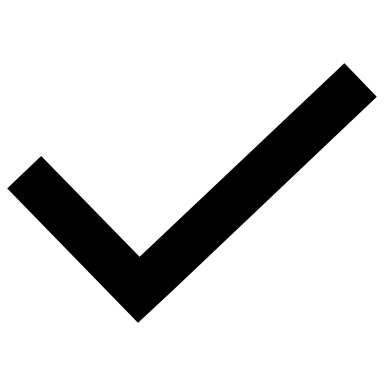 | 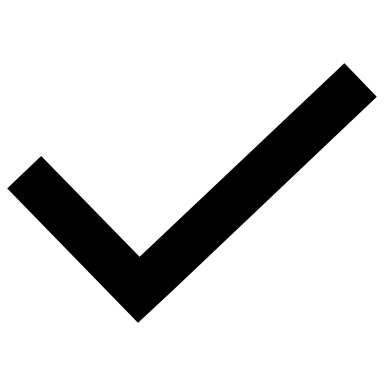 | 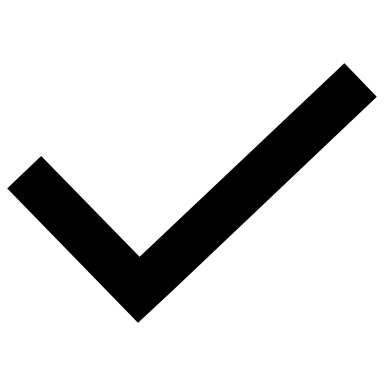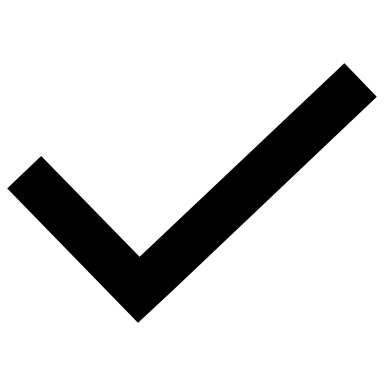 | 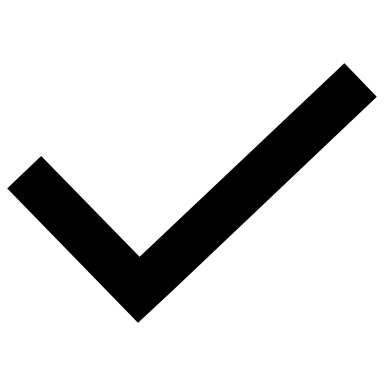 | 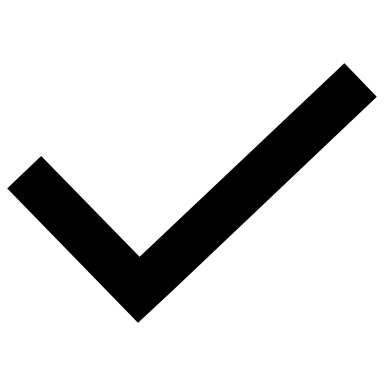 | 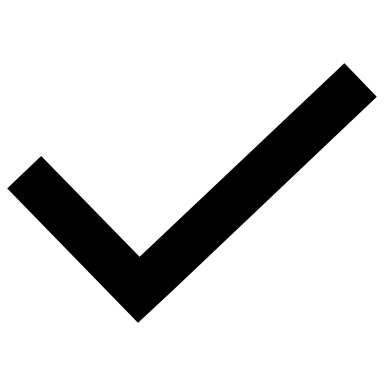 | 9 |

**Table E1.** Newcastle Ottawa Quality Assessment Scale for Cohort Studies. Maximum score 9 (4 for selection, 2 for comparability, and 3 for outcome).


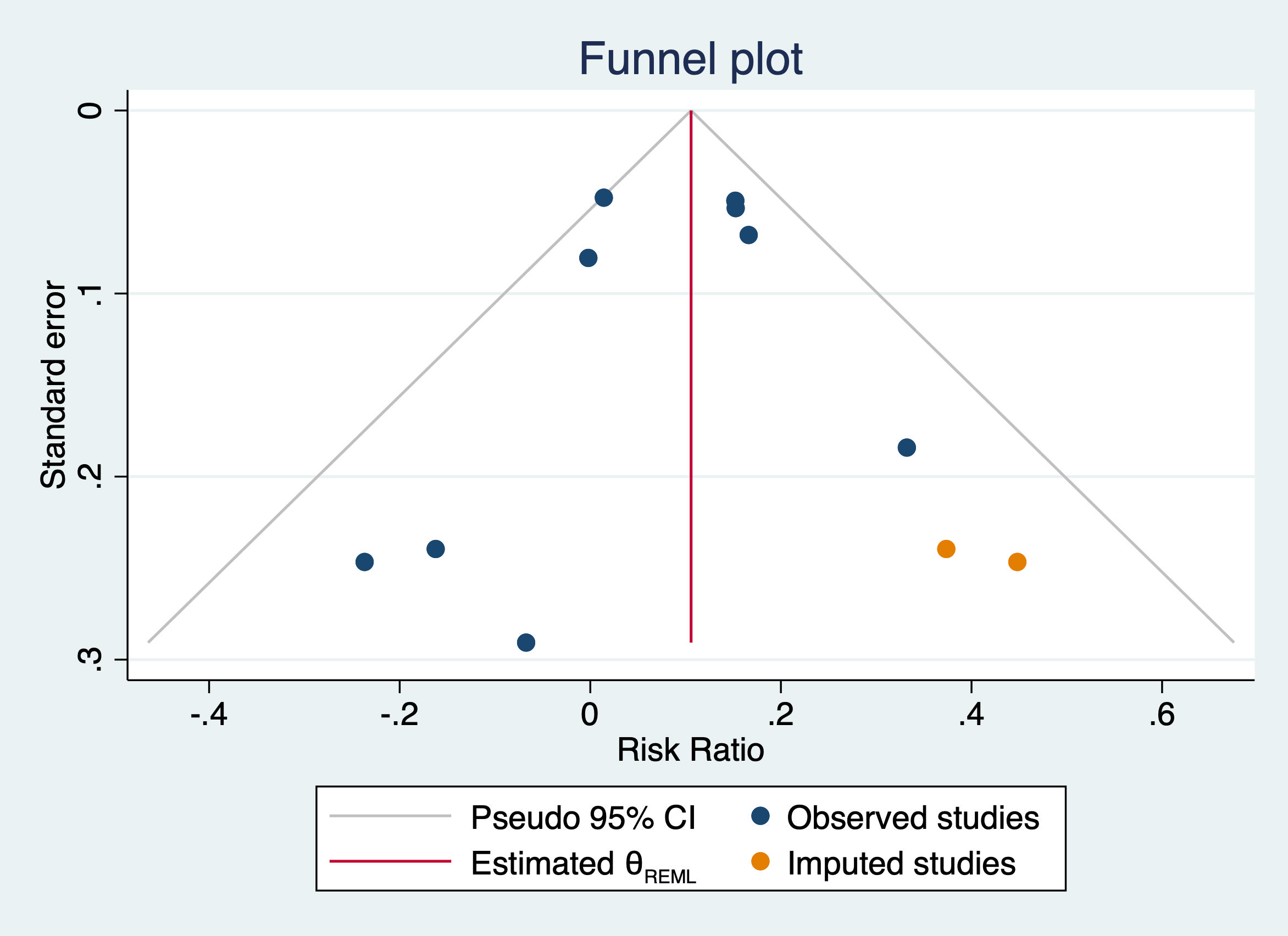


**Figure E1.** Trim-fill approach showing corrected publication bias. Blue circles represent original studies and orange circles represent added studies to account for publication bias.

|  | **Radiation side** | **Events/Total*** | **Time at Risk  (person-months)** | **Model 5 aHR (95%CI, P-value)** |
| --- | --- | --- | --- | --- |
| Total Population (n=243) | Right (n=125) | 11/243 | 973.56 | Reference |
|  | Left (n=118) |  |  | 0.04 (0.00-6.68, p=0.211) |

**Table E2.** Model 5: age, race, diabetes, hypertension, hypercholesterolemia, BMI, smoking status, CKD, prior CAD, cancer stage, type of surgery, radiation dose, endocrine therapy, immunotherapy, chemotherapy, use of HER2, and DIBH.
